# Mid-level healthcare workers knowledge on non-communicable diseases in Tanzania: a district-level pre-and post-training assessment

**DOI:** 10.1186/s12913-024-11078-w

**Published:** 2024-05-09

**Authors:** Peter Karoli, Mary Mayige, Gibson Kagaruki, Amani Mori, Edgar Macha, Reuben Mutagaywa, Arafa Momba, Harrieth Peter, Ritha Willilo, Pilly Chillo, Aidan Banduka, Bruno Sunguya, Kaushik Ramaiya, Edna Majaliwa, Stella Malangahe, Renatus Nyarubamba, Esther Mtumbuka, Elizabeth Mallya, Deogratias Soka, Sarah Urasa, Willfredius Rutahoile, Best Magoma, Emiliana Donald, David Mwenesano, Kajiru Kilonzo

**Affiliations:** 1 National Institute for Medical Research-Tanzania (NIMR), Dar es Salaam, Tanzania; 2 Muhimbili University of Health and Allied Sciences (MUHAS), Dar es Salaam, Tanzania; 3 The Tanzania NCD Alliance (TANCDA), Dar es Salaam, Tanzania; 4 Jakaya Kikwete Cardiac Institute (JKCI), Dar es Salaam, Tanzania; 5 Muhimbili National Hospital (MNH), Tanzania Muhimbili National Hospital (MNH),; 6 Clinton Health Access Initiative (CHAI), Dar es Salaam, Tanzania; 7 Tanzania Sickle Cell Disease Foundation (TANSCDF),, Sickle Cell Disease Foundation (TANSCDF), Dar es Salaam, Tanzania; 8 Kilimanjaro Christian Medical University College (KCMUCo), Moshi, Kilimanjaro Tanzania; 9 University of Bergen, Bergen, Norway; 10 Tanzania Diabetes Association (TDA), Dar es Salaam, Tanzania; 11 Benjamin Mkapa Hospital (BMH), Dodoma, Tanzania; 12 National Health Insurance Fund (NHIF), Dodoma, Tanzania; 13 Dodoma Regional Hospital, Dodoma, Tanzania; 14 Kilimanjaro Christian Medical Center (KCMC), Moshi, Kilimanjaro, Tanzania; 15 Kondoa Town Council, Kondoa, Dodoma, Tanzania; 16 Dodoma University, Dodoma, Tanzania

**Keywords:** Knowledge, Non-communicable diseases, Diabetes, Rheumatic heart disease, Sickle cell disease, PEN plus, Tanzania

## Abstract

**Introduction:**

Over the past two decades, Tanzania’s burden of non-communicable diseases has grown disproportionately, but limited resources are still prioritized. A trained human resource for health is urgently needed to combat these diseases. However, continuous medical education for NCDs is scarce. This paper reports on the mid-level healthcare workers knowledge on NCDs. We assessed the knowledge to measure the effectiveness of the training conducted during the initiation of a Package for Essential Management of Severe NCDs (PEN Plus) in rural district hospitals in Tanzania.

**Methods:**

The training was given to 48 healthcare employees from Dodoma Region’s Kondoa Town Council District Hospital. For a total of five (5) days, a fundamental course on NCDs featured in-depth interactive lectures and practical workshops. Physicians from Tanzania’s higher education institutions, tertiary university hospitals, research institutes, and medical organizations served as trainers. Before and after the training, a knowledge assessment comprising 28 questions was administered. Descriptive data analysis to describe the characteristics of the specific knowledge on physiology, diagnosis and therapy of diabetes mellitus, rheumatic fever, heart disease, and sickle cell disease was done using Stata version 17 (STATA Corp Inc., TX, USA).

**Results:**

Complete assessment data for 42 out of the 48 participants was available. Six participants did not complete the training and the assessment. The mean age of participants was 36.9 years, and slightly above half (52%) were above 35 years. Two-thirds (61.9%) were female, and about half (45%) were nurses. The majority had the experience of working for more than 5 years, and the average was 9.4 years (+/- 8.4 years). Overall, the trainees’ average scores improved after the training (12.79 vs. 16.05, *p* < 0.0001) out of 28 possible scores. Specifically, trainees’ average scores were better in treatment than in diagnosis, except for sickle cell disease (1.26 vs. 1.83). Most were not able to diagnose rheumatic heart disease (47.6% able) compared to diabetes mellitus (54.8% able) or sickle cell disease (64.3% able) at baseline. The proportion of trainees with adequate knowledge of the treatment of sickle cell disease and diabetes mellitus was 35% and 38.1%, respectively, and there was a non-statistical difference after training. Those working for less than 5 years had a higher proportion of adequate knowledge (30.8%) compared to their more experienced colleagues (6.9%). After the training, participants’ knowledge of NCDs increased by three times (i.e., aPR 3, 95% CI = 1.1, 1.5, and 6.0).

**Conclusion and recommendations:**

PEN Plus training improved the knowledge of healthcare workers at Kondoa Town Council District Hospital. Training is especially needed among nurses and those with a longer duration of work. Continuing education for human resources for health on the management of NCDs is highly recommended in this setting.

**Supplementary Information:**

The online version contains supplementary material available at 10.1186/s12913-024-11078-w.

## Introduction

The burden of non-communicable diseases (NCDs) is increasing globally [1]. By 2014, NCDs accounted for more than two-thirds of total global deaths and 40% of deaths in individuals younger than 70 years [2]. The majority (82%) of the premature deaths were from low- and middle‐income countries [1, 2]. According to the WHO, NCDs accounted for 33% of all deaths in Tanzania and they are projected to surpass the burden of deaths by infectious diseases in a few years to come [3]. Recent data shows that the burden of NCDs in Tanzania as expressed by Disability Adjusted Life Years (DALYs) is 41%, which means it has more than doubled compared to WHOs estimates of 19% in 1990 [4]. While the burden of NCDs has increased tremendously, a large share of healthcare resources i.e., 62% are still allocated to infectious diseases compared to NCDs (11%) [5]. This prompted the Tanzania National Institute for Medical Research (NIMR) to increase efforts and investment in NCD research as one of its key strategic areas in its institutional strategic plan 2019–2024 and National Health Research Agenda (NHRA-2018-2023) [6].

Tanzania has a population of 61.7 million people [7], with a decentralized health system that is organized in a pyramidal structure in tandem with the administrative system. Primary health facilities that include dispensaries, health centers, and district hospitals are the base—the latter function as the referrals for the health centers and dispensaries. Above the district hospitals, there are regional, zonal, specialized, and national referral hospitals. There are approximately 10,200 health facilities in Tanzania, and around 95% are primary health facilities mostly the health centers and dispensaries [8]. The Ministry of Health has the role of formulating policies, treatment guidelines, quality assurance, and resource mobilization while the President’s Office-Regional Administration and Local Government Authorities translate and implement policies from the district level downwards [9].

### Global NCDs challenge and the available healthcare services

Diabetes is one of the conditions that is increasing very fast globally, with the Sub-Saharan Africa being disproportionately affected [10]. The rate of diabetes in Africa reached 80% of the global rate which was 537 million people affected by diabetes in 2021 with the expectance to rise to783 million by 2045 [10]. However, these rates have been coupled with undiagnosed diabetes. In Tanzania, the rates of diabetes reached 12.3 in 2021 which is expected to rise more in the coming years[10]. Unfortunately, most of these data are in adults and more so in type 2 diabetes [10]. Less is known about Type 1 diabetes in sub–Saharan Africa, not to mention the children and youth. The IDF estimates up to 1.2 million children and adolescents have Type 1 diabetes, however, most of these die undiagnosed hence an unknown number of cases [10]. Of the data available in Tanzania, the incidence of type 1 diabetes is at 1.8–1.9/100,000 population [10]. This is a very low rate compared to 52.2 /100,000 population in Scandinavian countries [10, 11]. Some of the reasons for this low incidence rate are Low Knowledge in both the community and health care providers, high mortality due to misdiagnosis by health care providers, low public awareness, and myths about diabetes in children together with mismanagement due to lack of knowledge [12, 13].

While hypertension has for a long time been the focus of NCD programs in Tanzania, Rheumatic Heart Disease (RHD) is the third most common cause of heart failure (12%) after hypertensive heart disease (45%) and dilated cardiomyopathy (28%) [14]. : In the current practices in Tanzania, most of the NCDs, like RHD, are clinically diagnosed at primary health care facilities, including district-level facilities. Thereafter, all clinically diagnosed cases are given referral to the regional and/or zonal (tertiary level) facilities for further investigation (confirmation) and treatment. Contrary to PEN Plus, the project has provided laboratory equipment and supplies to ensure that all diagnosis and treatment are completed at district-level facilities to mitigate disease complications and individual costs, and only the already complicated cases would be given referrals for specialised care. For instance, patients with RHD seek medical attention when already in the late stages of their illness, hence presenting with complications (heart failure, infective endocarditis, atrial fibrillation, pulmonary hypertension, stroke, etc.). In 2010, 48% of patients who underwent open heart surgery at the Jakaya Kikwete Cardiac Institute (the national cardiac hospital) were due to RHD and the majority of these patients were young (mean age 19.4 ± 12.3 years) [15]. The management of these patients at late stages is associated with high-cost implications at the family level as well as in the health system leading to poor treatment outcomes including disability and death. The overall mortality rate among all patients undergoing cardiac surgery at JKCI was 13.3% of which 64.3% were in patients who underwent mitral valve repair [15].

In 2010, WHO introduced the Package of Essential NCD Interventions (WHO PEN) to support the integration of case management for common NCDs such as cardiovascular heart diseases, diabetes, and chronic respiratory diseases at primary health facilities in resource-limited settings [16]. By 2020, more than 30 countries including Tanzania had introduced the PEN program in their healthcare system. However, there were gaps in the management of severe NCDs particularly at primary healthcare facilities. In 2019, WHO AFRO introduced the PEN-Plus, which was an extension of the PEN strategy to address severe NCDs through integrated outpatient clinics at first-level referral health facilities [17]. On 23rd August 2022, the 47 WHO-AFRO member states adopted the PEN Plus strategy [17]. The objectives of the PEN Plus strategy are (1) to strengthen the availability of, and access to quality services for severe NCDs (2) To improve the capacity of the healthcare workers to provide integrated care for severe NCDs; (3) To improve the availability of essential medicines and equipment for the management of severe NCDs and (4). To support monitoring and evaluation and research on PEN-Plus interventions [17].

The available NCD services in Tanzania do not match the current burden and are not uniformly well-prepared for the projected increase; there is high variability between rural and urban areas as far as availability, access, and presence of trained personnel. Health care personnel’s training in new topics and skills ensures high-quality health education and services, significantly influencing the success of health programs and services, as demonstrated by published data [23, 28]. A study conducted in Ethiopia, found that mid-level healthcare workers, whether employed in field or hospital settings, lacked adequate skills, underscoring the need for more on job training [29].

Significant improvements have been made in diabetes care, however, through the National Diabetes Programme where specific interventions were implemented contributing to the establishment of diabetes / NCD services at all National, Zonal, Regional & District Hospitals. Late presentation and ensuing complications for NCDs pose an urgent need for early diagnosis and treatment through the creation of community awareness, enabling healthcare workers, and improving detection capacity at lower-level health facilities.

In collaboration with healthcare facilities in the country as well as the Ministry of Health, the National Institute for Medical Research is leading the efforts to generate evidence to support roll out of PEN PLUS model of care for severe NCDs at first-level (district) hospitals and support the provision of PEN services for other NCDs in the primary care facilities. This demonstration project will be embedded within the existing health system and infrastructure so that it can be sustained over time. We expect to generate robust data on the organization of care at first-level hospitals for underrepresented groups in cardiovascular care (RHD), type 1 diabetes, and sickle cell disease of which the goal is for improvement of care and potential national scale-up of the interventions.

Therefore, this study reports on the mid-level healthcare workers knowledge on NCDs. We assessed the knowledge to measure the effectiveness of the training conducted during the initiation of a Package for Essential Management of Severe NCDs (PEN Plus) in rural district hospitals in Tanzania.

## Methods

### Study design and setting

In March 2023, HCWs from Kondoa Town Council District Hospital participated in a pre-post (before and after) training assessment. Kondoa Town Council District Hospital is a primary health care facility in rural Dodoma located centrally, in Tanzania. The hospital serves an estimated population of 226,154 through its outpatient and inpatient services. The hospital has a capacity of 200 beds and averages 50–70 occupancy each day. This study site was purposely selected as researchers are in the process of implementing PEN Plus and have already established a relationship with the local government, hospital administration, and HCWs.

### Study population and sample size

The study comprised all PEN-Plus-trained healthcare staff from Kondoa Town Council District Hospital. The hospital administration picked these healthcare workers based on their needs to meet the facility’s goal of improving NCD care. A total of 48 healthcare employees from various cadres were asked to attend a one-week training on PEN Plus, including medical doctors, assistant medical doctors, clinical officers, and pharmacy technicians. However, 42 out of 48 completed the training and had both pre-post data thus resulting in a response rate of 87.5%.

### Data collection process and tools

HCWs’ clinical knowledge of the three NCDs: diabetes, sickle cell disease, rheumatic fever, and heart disease (cardiovascular illness) was assessed using structured self-administered questionnaires in the form of a pre-post-test exam. The baseline assessment of knowledge on NCD questions were adapted from Malawi PEN Plus training materials. The tool comprised 28 multiple-choice questions, ten [10] of which assessed knowledge of diabetes, eight [8] of which assessed knowledge of rheumatic fever and heart disease (cardiovascular illness), and ten [10] of which assessed knowledge of sickle cell disease. The questions were mostly focused on the diagnosis and treatment of the three selected NCDs; they were designed and given in English (Appendix).

### Study variables

The study had one main outcome variable, which is the targeted PEN Plus NCDs knowledge scores. For all conditions; diabetes, cardiovascular disease, and sickle cell diseases, we used Bloom’s cut-off point scores to categorize participants as having good or poor knowledge [30] as summarised in Table 1 below. For instance, the max scores for overall NCDs were 28, and HCW was considered to have adequate knowledge if scored 16 or above. Independent variables were socio-demographic factors including age, gender, professional affiliation, and working experience that were considered to affect knowledge.

**Table 1:** Shows the cut off points used to categorize knowledge of healthcare workers

Disease	Disease domain	Max/possible scores	Expected/best scores (adequate)
Diabetes Mellitus	Physiology	1	1
	Diagnosis	4	2+
	Treatment	5	3+
Heart failure	Diagnosis	3	2+
	Physiology	2	1+
Rheumatic Heart Diseases	Diagnosis	1	1
	Treatment	2	1+
Sickle Cell Disease	Diagnosis	3	2+
	Complications	1	1
	Risk factors	2	1+
	Treatment	4	2+
Overall NCDs	All three diseases	28	16+

### Data collection, management, and analysis

The pre and post-test questionnaires were filled on Open Data Kit (ODK) software which was programmed and installed in the tablets. Before downloading the data from the cloud server, all tablets were checked to ensure that all completed questionnaires had been uploaded to the server. The complete dataset was downloaded in a .csv (Comma Separated Value) format. Data downloaded from the server were then exported from Excel to Stata version 17 (STATA Corp Inc., TX, USA) for cleaning and analysis. Data analysis started with running descriptive statistics to describe the characteristics of the data set. This included running the univariate analysis on frequency and percentage response distributions and measures of central tendency and dispersion. Later, we conducted inferential analysis using appropriate methods (e.g., Chi-squares and Modified Poison logistic regression). Modified Poison Logistic Regression Models was opted since the proportion of HCWs with adequate knowledge was greater than 10%, hence this model is expected to fit well the outcome and explanatory variables as opposed to the classical logistic regression model. Classical Logistic Regression overestimates odd ratios when the outcome of interest is ≥ 10% [18, 19]. Bivariate analysis assessed the difference in the proportions of target indicators using a Chi-square test for categorical variables. We reported both the Unadjusted Prevalence Ratio (uPR) and the Adjusted Prevalence Ratio (aPR). Association between dependent and independent variables was considered significant if *p*-value < 0.05.

## Results

### Demographic profile

The mean age of the participants was 36.9 years. However, more than half (52.4%) were above 35 years. About 62% of the participants were female and 45% and 24% were nurses and medical doctors, respectively. Their working experience averaged 9.4 years but more than two-thirds i.e., 69% had experience working for more than 5 years (Table 2).

**Table 2:** Demographic characteristics of study participants

Variable	Category	Number	Percentage
Mean age in years (SD)		36.9(8.8)	
Age group above 35 years		22	52.4
Female Gender		26	61.9
Cadre	Doctors	10	23.8
	Nurses	19	45.2
	Others	13	31
Mean years of working (SD)	Mean (SD)	9.4(8.4)	
Working experience category	5 + yrs	29	69

### NCD knowledge

The proportion of healthcare workers who answered correctly on diabetes mellitus, rheumatic heart diseases, and sickle cell disease-specific questions on basic physiology, diagnosis, and treatment during pre and post-tests are presented in Tables 3, 4 and  5. The results show that trainees’ average scores were significantly better in the post-test phase than the pre-test phase except for sickle cell disease on treatment (1.26 vs. 0.19, *p* = 0.009). Most were not able to diagnose rheumatic heart disease (47.6% able) compared to diabetes mellitus (54.8% able) or sickle cell disease (64.3% able) at baseline. The proportion of trainees with adequate knowledge of the treatment of sickle cell disease and diabetes mellitus was 35% and 38.1%, respectively, and there was a non-statistical difference after training (Table 6). Those working for less than 5 years had a higher proportion of adequate knowledge (30.8%) compared to the more experienced colleagues (6.9%) (Table 7). After the training, participants’ knowledge of NCDs increased by 3 times (i.e. aPR 3, 95% CI = 1.1-1.5-6.0) Table 8.

**Table 3:** Proportion of healthcare workers who answered correctly on diabetes mellitus during pre and post-tests

Questions		Correct answer	Pre-test, n(%)	Post-test, n(%)	*p*-values
Diagnosis					
A 25-year-old man presents to the OPD with weight loss and getting up at night often to urinate. You suspect diabetes. What test/s can you use to diagnose diabetes?		Random blood glucose & Haemoglobin A1c test	11.9	59.5	<0.001
A 45-year-old was the previous week. On routine screening her BMI was 30 kg/m2 and blood pressure is 150/90. She denies any symptoms of diabetes. Her finger stick blood sugar (random) was 210 mg/dl (11.7 mmol/L). Does this patient have diabetes?		Maybe, but only if we repeat her random glucose and it’s ≥ 200 mg/dl.	42.9	35.7	0.503
A 30-year-old girl with Type I Diabetes presents to the OPD. She has been on insulin therapy since her diagnosis at age 25. She is recovering from a diarrheal illness and has not been eating much. She awoke this morning feeling very anxious, weak, and dizzy. She has a heart rate of 110 bpm, blood pressure 110/70, oxygen saturation 99%. Malaria test is negative. What is your next step?		Check random blood glucose for hypoglycaemia	85.7	97.6	0.048
Which statement is true		If symptomatic, a single FBS ≥ 126 mg/dL (7.0 mmol/L) is diagnostic of diabetes	23.8	33.3	0.334
Physiology					
What is the effect of insulin on the blood glucose level?		Causes a decrease in the blood glucose level	76.2	92.9	0.035
Treatment					
A 70-year-old woman with long-standing diabetes comes to the integrated chronic care clinic. She takes metformin 1000 mg twice daily, and enalapril 20 mg daily. She has no current complaints. She gets an I-STAT and you see that her Creatinine is 2.8. What changes, if any do you make to her medications?		Stop the enalapril	19.1	19.1	1.000
A 54-year-old woman with diabetes presents to OPD with diarrhoea and is confused and has difficulty staying awake. Blood pressure is 110/78, heart rate is 100, oxygen saturation is 99%. Blood glucose is taken and returns at 560 mg/dl. You prepare for regular glucose checks and insulin administration. What other parts of treatment of HONK are critical here?		Normal saline boluses and potassium repletion	81	83.3	0.776
A 78-year-old man presents to the chronic care clinic. He has been receiving treatment for her diabetes and hypertension. He takes metformin 1000 g twice daily, enalapril 20mg daily, and amlodipine 5mg daily. He smokes cigarettes daily. You calculate that he has a 30-40% cardiovascular risk. After you advise him on quitting smoking, what is your next step?		Start aspirin and simvastatin	33.3	33.3	1.000
A 45-year-old woman with type 2 diabetes presents to the chronic care clinic for regular follow-up. She was last seen 6 months ago and has had good control of her sugar levels on metformin 500mg twice daily. She has a heart rate of 80, blood pressure of 160/94. Random glucose measurement is 130. What medication would you like to start?		Enalapril 10mg daily	19.1	19.1	1.000
A 56-year-old man has recently been diagnosed with type 2 diabetes. Today, his heart rate is 70, his blood pressure is 120/80, and his BMI is 32. What things can you discuss with him that will improve his diabetes?		Increasing the amount of daily exercise, advise on eating more vegetables, tell him to take his medications regularly	78.6	81	0.786
Category	Stem of multiple-choice question on Diabetes Mellitus	Correct answer	Pre-test, n(%)	Post-test, - n(%)	P-values
Physiology	What is the effect of insulin on the blood glucose level?	Causes a decrease in the blood glucose level.	32(76.2)	39(92.9)	0.035
Diagnosis	A 25-year-old man presents to the OPD with weight loss and getting up at night often to urinate. You suspect diabetes. What test/s can you use to diagnose diabetes?	Random blood glucose and Haemoglobin A1c test	5(11.9)	25(59.5)	<0.001
Diagnosis	A 45-year-old was the previous week. On routine screening her BMI was 30 kg/m2 and blood pressure is 150/90. She denies any symptoms of diabetes. Her finger stick blood sugar (random) was 210 mg/dl (11.7 mmol/L). Does this patient have diabetes?	Maybe, but only if we repeat her random glucose and it’s ≥ 200 mg/dl.	18(42.9)	15(35.7)	0.503
Treatment	A 70-year-old woman with long-standing diabetes comes to the integrated chronic care clinic. She takes metformin 1000 mg twice daily, and enalapril 20 mg daily. She has no current complaints. She gets an I-STAT and you see that her Creatinine is 2.8. What changes, if any, do you make to her medications?	Stop the metformin	8(19.1)	16(19.1)	1.000
Diagnosis	A 30-year-old girl with Type I Diabetes presents to the OPD. She has been on insulin therapy since her diagnosis at age 25. She is recovering from a diarrheal illness and has not been eating much. She awoke this morning feeling very anxious, weak, and dizzy. She has a heart rate of 110 bpm, blood pressure 110/70, oxygen saturation 99%. The Malaria test is negative. What is your next step?	Check random blood glucose for hypoglycaemia	36(85.7)	41(97.6)	0.048
Treatment	A 54-year-old woman with diabetes presents to OPD with diarrhoea and is confused and has difficulty staying awake. Blood pressure is 110/78, heart rate is 100, oxygen saturation is 99%. Blood glucose is taken and returns at 560 mg/dl. You prepare for regular glucose checks and insulin administration. What other parts of treatment of HONK are critical here?	Normal saline boluses and potassium repletion	34(81.0)	35(83.3)	0.776
Treatment	A 78-year-old man presents to the chronic care clinic. He has been receiving treatment for her diabetes and hypertension. He takes metformin 1000 g twice daily, enalapril 20 mg daily, and amlodipine 5mg daily. He smokes cigarettes daily. You calculate that he has a 30-40% cardiovascular risk. After you advise him on quitting smoking, what is your next step?	Start aspirin and simvastatin	14(33.3)	14(33.3)	1.000
Treatment	A 45-year-old woman with type 2 diabetes presents to the chronic care clinic for regular follow-up. She was last seen 6 months ago and has had good control of her sugar levels on metformin 500mg twice daily. She has a heart rate of 80, blood pressure of 160/94. Random glucose measurement is 130. What medication would you like to start?	Enalapril 10mg daily	8(19.1)	8(19.1)	1.000
Treatment	A 56-year-old man has recently been diagnosed with type 2 diabetes. Today, his heart rate is 70, his blood pressure is 120/80, and his BMI is 32. What things can you discuss with him that will improve his diabetes?	Increasing the amount of daily exercise, advise on eating more vegetables and tell him to take his medications regularly	33(78.6)	34(81.0)	0.786
Diagnosis	Which statement is true regarding diagnosis of Diabetes mellitus	If symptomatic, a single FBS ≥ 126 mg/dL (7.0 mmol/L) is diagnostic of diabetes	10(23.8)	14(33.3)	0.334

**Table 4:** Proportion of healthcare workers who answered correctly on rheumatic heart disease and its complications during pre and post-tests

Domain	Stem of multiple-choice question on Rheumatic heart disease and its complications	Correct answers	Pre-test, *n*(%)	Post-test, *n*(%)	*P*-values
Diagnosis	One of the following values for the ejection fraction (EF) does NOT typically represent systolic heart failure	60%	13(30.95)	8(19.05)	0.208
Physiology	Choose the correct order of blood flow through the right heart	Vena cava, right atrium, tricuspid valve, right ventricle	22(52.38)	21(50)	0.827
Physiology	Choose the correct order of blood flow through the left heart	Left atrium, mitral valve, left ventricle, aortic valve	13(30.95)	31(73.81)	< 0.001
Diagnosis	Which of the following is a cause of a heart failure exacerbation?	Non-adherence to medications, Change in diet, Acute Illness, Anaemia	21(50)	32(76.19)	0.013
Diagnosis	A 32-year-old woman comes to the clinic. She was doing well until 2 months ago, when she started experiencing some difficulty breathing while going up the hill. She can perform her daily activities. She was pregnant and gave birth to her son 3 months ago. You diagnose her with postpartum cardiomyopathy. Her NYHA classification is	Class I/II	32(76.19)	31(73.81)	0.801
Diagnosis	Rheumatic heart disease is caused by which of the following?	Repeated infections with Group A strep leading to immune response and damage to the heart valves, which can be prevented with penicillin.	20(47.62)	36(85.71)	< 0.001
Treatment	Which of the following patients should be treated with penicillin for their pharyngitis for primary prophylaxis to prevent rheumatic fever?	A 14 yo female with exudate of throat on exam, fever, and no cough	13(30.95)	29(69.05)	< 0.001
Treatment	You diagnose a 14-year-old patient with rheumatic heart disease. Which of the following antibiotics and duration is indicated in this patient?	Oral Penicillin V daily or benzathine penicillin G monthly, for at least 10 years	9(21.43)	21(50)	0.006

**Table 5:** Proportion of healthcare workers who answered correctly on rheumatic heart disease and its complications during pre and post-tests

Domain	Stem of multiple-choice question on Rheumatic heart disease and its complications	Correct answers	Pre-test, *n*(%)	Post-test, *n*(%)	*P*-values
Diagnosis	One of the following values for the ejection fraction (EF) does NOT typically represent systolic heart failure	60%	13(30.95)	8(19.05)	0.208
Physiology	Choose the correct order of blood flow through the right heart	Vena cava, right atrium, tricuspid valve, right ventricle	22(52.38)	21(50)	0.827
Physiology	Choose the correct order of blood flow through the left heart	Left atrium, mitral valve, left ventricle, aortic valve	13(30.95)	31(73.81)	< 0.001
Diagnosis	Which of the following is a cause of a heart failure exacerbation?	Non-adherence to medications, Change in diet, Acute Illness, Anaemia	21(50)	32(76.19)	0.013
Diagnosis	A 32-year-old woman comes to the clinic. She was doing well until 2 months ago, when she started experiencing some difficulty breathing while going up the hill. She can perform her daily activities. She was pregnant and gave birth to her son 3 months ago. You diagnose her with postpartum cardiomyopathy. Her NYHA classification is	Class I/II	32(76.19)	31(73.81)	0.801
Diagnosis	Rheumatic heart disease is caused by which of the following?	Repeated infections with Group A strep leading to immune response and damage to the heart valves, which can be prevented with penicillin.	20(47.62)	36(85.71)	< 0.001
Treatment	Which of the following patients should be treated with penicillin for their pharyngitis for primary prophylaxis to prevent rheumatic fever?	A 14 yo female with exudate of throat on exam, fever, and no cough	13(30.95)	29(69.05)	< 0.001
Treatment	You diagnose a 14-year-old patient with rheumatic heart disease. Which of the following antibiotics and duration is indicated in this patient?	Oral Penicillin V daily or benzathine penicillin G monthly, for at least 10 years	9(21.43)	21(50)	0.006

**Table 6:** Average scores and percentage of training participants who scored the expected scores on different NCDs domains

Disease domain	Max scores	Average scores: Pre-test	Average scores: Post-test	*p*-values	Expected/ best scores	% scored the expected scores-		*p*-value
						Pre-test = 42(%)	Post-test = 42(%)	
*Diabetes Mellitus*								
Physiology	1	0.76	0.93	0.035	1	32(76.2)	39(92.9)	0.035
Diagnosis	4	1.64	2.26	0.002	2+	23(54.8)	34(81.0)	0.01
Treatment	5	2.31	2.36	0.828	3+	16(38.1)	19(45.2)	0.441
*Heart failure*								
Diagnosis	3	1.57	1.69	0.508	2+	42(100)	42(100)	NA
Physiology	2	0.83	1.24	0.01	1+	29(69.1)	34(81.0)	0.208
*Rheumatic Heart Diseases*								
Diagnosis	1	0.48	0.86	< 0.001	1	20(47.6)	36(85.7)	< 0.001
Treatment	2	0.52	1.19	< 0.001	1+	17(40.5)	34(81.0)	< 0.001
*Sickle Cell Disease*								
Diagnosis	3	1.86	1.83	0.897	2+	27(64.3)	30(71.4)	0.483
Complications	1	0.88	0.92	0.463	1	37(88.1)	39(92.9)	0.457
Risk factors	2	0.67	0.91	0.135	1+	23(54.8)	28(66.7)	0.264
Treatment	4	1.26	0.19	0.009	2+	15(35.7)	23(54.8)	0.079
All three diseases	28	12.79	16.05	< 0.001	16+	6(14.3)	18(42.9)	0.004

**Table 7:** Association between adequate knowledge and demographic profile of the participants: Pre-test and Post-test analysis

Variable	Category	*N*	Pre-test		Post-test	
			Adequate knowledge, *n*(%)	*P*-value	Adequate knowledge, *n*(%)	*P*-value
Age group	< 35yrs	20	1(5.0)	0.101	11(55.0)	0.129
	35 + yrs	22	5(22.7)		7(31.8)	
Gender	Male	16	5(31.3)	0.014	12(75.0)	0.001
	Female	26	1(3.9)		6(23.1)	
Cadre	Doctors	10	4(40.0)	0.028	9(90.0)	0.001
	Nurses	19	1(5.3)		3(15.8)	
	Others	13	1(7.7)		6(46.2)	
Working experience	< 5yrs	13	4(30.8)	0.041	9(69.2)	0.021
	5 + yrs	29	2(6.9)		9(31.0)	

**Table 8:** Effect of training on adequate NCDs knowledge of the participants: Adjusted for carder, gender and working experience

Variable	uPR,95%CI	*P*-value	aPR,95%CI	*P*-value
Phase				
Pre-test	Ref		Ref	
Post-test	3.0(1.3–6.8)	0.009	3.0(1.1-1.5-6.0)	0.002

*uPR *unadjusted prevalence ratio,*  aPR a*djusted prevalence rati*o, CI c*onfidence interval

## Discussion

It is well established that the burden of NCDs is growing rapidly in low- and middle-income countries (LMICs), including in sub-Saharan Africa [1]. Despite this, the resources allocated for the prevention and management of NCDs are often insufficient, with a large share of the healthcare budget directed towards communicable diseases like malaria, tuberculosis, and HIV/AIDS [20]. For example, in Tanzania, infectious diseases accounted for 63% of the total health expenditure while NCDs and injuries accounted for 10% and 2%, respectively [21]. Expenditure on NCDs as a proportion of Total Health Expenditure in Tanzania has not changed since 2015 [22]. This lack of resources is compounded by a shortage of trained healthcare workers and providing them with continuous or job training, which is a significant problem in many sub-Saharan African countries [23]. We see most communicable diseases being more funded by donors, hence there is a need for increasing internal resources for non-communicable diseases since planners have control over the internal sources as opposed to the external ones.

Children and young adults among the world’s poorest communities in sub-Saharan Africa and Asia carry around half the burden of Non-Communicable Diseases and Injuries [24]. Severe NCDs such as congenital and rheumatic heart diseases, diabetes type 1, and sickle cell disease which are most prevalent in these age groups are responsible for 150,000 preventable deaths [25]. These conditions can be addressed through improved access to cheap and cost-effective treatments that are widely available in LMICs. Unfortunately, the diagnosis and treatment of severe NCDs in these countries are restricted to tertiary health facilities that are mostly found in urban areas [17]. On the contrary, the majority of the people in LMICs reside in rural areas and often rely upon the primary healthcare facilities where diagnosis and treatment of severe NCDs are currently unavailable due to poor infrastructure and the lack of trained human resources. To address the shortage of skilled HCWs to manage severe NCDs at lower-level facilities, the PEN Plus team in Tanzania held a 5-day comprehensive training program called NCDs foundational training to facilitate the execution of the Package for essential NCDs (PEN Plus) management among the HCWs at Kondoa Town council Hospital in Tanzania’s. For five days, foundational training included in-depth interactive lectures and practical workshops. The trainers were physicians from Tanzanian training institutions, tertiary hospitals, research institutes, and the Tanzania Medical Association. Before and after the training, we performed a knowledge assessment comprising twenty-eight (28) questions about the physiology, diagnosis, and therapy of diabetes mellitus, rheumatic fever/heart disease, and sickle cell disease. The findings showed that the HCWs had low NCD knowledge, however, post-test results indicated that they are trainable, hence there is a need for investing in continuous training to equip these staff with adequate skills and knowledge to address the current surge of NCDs which is responsible for about 33% of all mortalities in the country3.

Before the training, the majority of the trainees did not have adequate knowledge about the diagnosis and treatment of rheumatic heart diseases, diabetes type 1, and sickle cell. However, after 5 days of intensive training, the knowledge of the healthcare workers improved by three times. Similar findings have been documented in Malawi, whereby a 7-day training on the diagnosis, treatment, and counselling for complex NCDs led to a significant increase in the knowledge of mid-level healthcare workers [26]. The proportion of trainees with knowledge of treatment for sickle cell disease and diabetes mellitus was the least and showed a non-statistical difference after training. Those with less than 5 years of work experience had a higher proportion of adequate knowledge compared to more experienced colleagues.

The findings of this study have important implications for healthcare policy and practice in sub-Saharan Africa. There is a need for ongoing training and education on NCDs, particularly among nurses and those with longer working experience. Continuing education programs that are tailored to the needs of healthcare workers in resource-limited settings should be prioritized. These programs can help to improve the quality of care for NCDs and could reduce the burden of NCDs in sub-Saharan Africa.

## Limitation

In terms of the study methodology, the use of a pre-and post-training knowledge assessment is a commonly used approach to evaluate the effectiveness of training programs [27, 28]. However, the sample size of 42 participants in just one facility may limit the generalizability of the study findings. Future studies should consider a much larger sample size of participants and the inclusion of a wide range of healthcare workers from different health facilities in the region. The attitudes and practices of HCW were not captured in this study. The absent data may have provided insightful information to readers about the gaps that now exist among healthcare providers.

## Conclusion

A five-day comprehensive PEN Plus training improved the knowledge of healthcare workers to manage severe NCDs at Kondoa District Hospital in Tanzania. The training was mostly effective among healthcare workers with longer duration of work exceeding five years, medical doctors, and male gender. Therefore, continuing education of district-level healthcare workers on the management of NCDs is highly recommended in this type of setting.

### Supplementary Information

Supplementary Material 1: Data collection tool a. Questionnaire b. Answer sheet.

## Data Availability

All data are freely accessible to the corresponding author with a valid data transfer agreement form from Tanzanian research regulatory bodies.
