# Supplementary material for: Mid-level healthcare workers knowledge on non-communicable diseases in Tanzania: a district-level pre-and post-training assessment
Source: BMC Health Serv Res. 2024 May 9;24:612. doi: 10.1186/s12913-024-11078-w (PMC11084094; doi:10.1186/s12913-024-11078-w)
Supplement: Supplementary file 1 — Supplementary Material 1: Data collection tool a. Questionnaire b. Answer sheet. [file 12913_2024_11078_MOESM1_ESM.docx]

**Healthcare workers knowledge on non-communicable diseases.**

**NCD Pre and Post training Exam**

1. What is the effect of insulin on the blood glucose level?
   1. Causes a decrease in the blood glucose level.
   2. Causes an increase in the blood glucose level.
   3. Does not affect the blood glucose level.
   4. A and B are true responses
   5. C with an additional explanation

1. A 25-year-old man presents to the OPD with weight loss and getting up at night often to urinate. You suspect diabetes. What test/s can you use to diagnose diabetes?
   1. Urinalysis
   2. Random blood glucose
   3. Hemoglobin A1c test
   4. Sputum sample
   5. Both B and C

1. A 45-year-old was the previous week. On routine screening her BMI was 30 kg/m2 and blood pressure is 150/90. She denies any symptoms of diabetes. Her finger stick blood sugar (random) was 210 mg/dl (11.7 mmol/L). Does this patient have diabetes?
   1. Yes
   2. No
   3. Maybe, but only if we repeat her random glucose and it’s ≥ 200 mg/dl.
   4. Maybe, but it depends on her weight

1. A 70-year-old woman with long-standing diabetes comes to the integrated chronic care clinic. She takes metformin 1000 mg twice daily, and enalapril 20 mg daily. She has no current complaints. She gets an I-STAT and you see that her Creatinine is 2.8. What changes, if any do you make to her medications?
   1. None, her metformin protect her kidneys
   2. Stop the enalapril
   3. Stop the metformin
   4. Stop both the metformin and enalapril

1. A 30-year-old girl with Type I Diabetes presents to the OPD. She has been on insulin therapy since her diagnosis at age 25. She is recovering from a diarrheal illness and has not been eating much. She awoke this morning feeling very anxious, weak, and dizzy. She has a heart rate of 110 bpm, blood pressure 110/70, oxygen saturation 99%. Malaria test is negative. What is your next step?
2. Give her diazepam for panic attack
3. Check I-STAT for kidney failure
4. Check random blood glucose for hypoglycemia
5. Give amoxycillin for suspected pneumonia

1. A 54-year-old woman with diabetes presents to OPD with diarrhea and is confused and has difficulty staying awake. Blood pressure is 110/78, heart rate is 100, oxygen saturation is 99%. Blood glucose is taken and returns at 560 mg/dl. You prepare for regular glucose checks and insulin administration. What other parts of treatment of HONK are critical here?
   1. Normal saline boluses and potassium repletion
   2. Lisinopril and metformin
   3. Aspirin and amlodipine
   4. Diazepam

1. A 78-year-old man presents to the chronic care clinic. He has been receiving treatment for her diabetes and hypertension. He takes metformin 1000 g twice daily, enalapril 20mg daily, and amlodipine 5mg daily. He smokes cigarettes daily. You calculate that he has a 30-40% cardiovascular risk. After you advise him on quitting smoking, what is your next step?
   1. Start aspirin and simvastatin
   2. Start furosemide
   3. Increase metformin to 2g twice daily
   4. No medication changes

1. A 45-year-old woman with type 2 diabetes presents to the chronic care clinic for regular follow-up. She was last seen 6 months ago and has had good control of her sugar levels on metformin 500mg twice daily. She has a heart rate of 80, blood pressure of 160/94. Random glucose measurement is 130. What medication would you like to start?
   1. Increase metformin to 1g twice daily
   2. Amlodipine 5mg daily
   3. Enalapril 10mg daily
   4. Propranolol 40mg twic daily

1. A 56-year-old man has recently been diagnosed with type 2 diabetes. Today, his heart rate is 70, his blood pressure is 120/80, and his BMI is 32. What things can you discuss with him that will improve his diabetes?
   1. Increasing the amount of daily exercise
   2. Advise on eating more vegetables
   3. Tell him there is nothing he can do for his diabetes
   4. Tell him to take his medications regularly
   5. Choices A, B, and D

1. Which statement is true

- 1. If symptomatic, a single FBS **≥ 126 mg/dL (7.0 mmol/L)** is diagnostic of diabetes
  2. If asymptomaitic, a single FBS **≥ 126 mg/dL** (**7.0 mmol/L**) is diagnostic of diabetes
  3. If asymptomatic a single RBS of **≥ 200 mg/dL (11.1 mmol/L)** is diagnostic of diabetes
  4. If symptomatic a single RBS of **≥ 126 mg/dL (7.0 mmol/L)** is diagnostic of diabetes

1. One of the following values for the ejection fraction (EF) does NOT typically represent systolic heart failure
   1. 60%
   2. 35%
   3. 15%
   4. 5%
   5. None of the Above
2. Choose the correct order of blood flow through the right heart
   1. Right atrium, vena cava, right ventricle, tricuspid valve
   2. Right ventricle, right atrium, vena cava, tricuspid valve
   3. Vena cava, right atrium, tricuspid valve, right ventricle
   4. Vena cava, pulmonary artery, tricuspid valve

1. Choose the correct order of blood flow through the left heart
   1. Left atrium, mitral valve, left ventricle, aortic valve
   2. Aortic valve, left ventricle, mitral valve, left atrium
   3. Left ventricle, mitral valve, aortic valve, left atrium
   4. Left atrium, aortic valve, left ventricle, mitral valve

1. Which of the following is a cause of a heart failure exacerbation?
   1. Non-adherence to medications
   2. Change in diet
   3. Acute Illness
   4. Anemia
   5. All of the Above

1. A 32-year old woman comes to the clinic. She was doing well until 2 months ago, when she started experiencing some difficulty breathing while going up the hill. She can perform her daily activities. She was pregnant and gave birth to her son 3 months ago. You diagnose her with postpartum cardiomyopathy. Her NYHA classification is
   1. Class I/II
   2. Class III
   3. Class IV
   4. Class V

1. Rheumatic heart disease is caused by which of the following?
   1. It is a congenital form of heart disease that a patient is born with
   2. High blood pressure that is untreated
   3. Untreated recurrent tuberculosis infection
   4. Repeated infections with Group A strep leading to immune response and damage to the heart valves, which can be prevented with penicillin.
2. Which of the following patients should be treated with penicillin for their pharyngitis for primary prophylaxis to prevent rheumatic fever?
   1. A 45 yo male with sore throat, no fever, and a cough
   2. A 14 yo female with exudate of throat on exam, fever, and no cough
   3. A 65 yo male with sore throat, cough, runny nose
   4. A 50 yo female with sore throat, no fever, and nasal congestion
3. You diagnose a 14-year-old patient with rheumatic heart disease. Which of the following antibiotics and duration is indicated in this patient?
   1. Cephalexin daily until age 21 years
   2. Oral Penicillin V daily or benzathine penicillin G monthly, for at least 10 years
   3. Bactrim daily for 7 days
   4. Oral Penicillin V daily or benzathine penicillin G monthly, until symptoms improve
4. Which of the following is a characteristic feature of sickle cell disease?

a) Abnormal hemoglobin production

b) Increased red blood cell count

c) Normal red blood cell shape

d) Low blood pressure

1. Which of the following is a common complication of sickle cell disease?

a) Hypertension

b) Migraine headaches

c) Anemia

d) Osteoporosis

1. What is the likelihood of inheriting sickle cell disease if both parents are carriers of the gene?
2. 25%
3. 50%
4. 75%
5. 100%
6. Which of the following can trigger a sickle cell crisis?

a) Cold weather

b) Exercise

c) Infection

d) All of the above

1. What is the primary symptom of sickle cell anemia?

a) Yellowing of the skin and eyes

b) Shortness of breath

c) Pain

d) Numbness and tingling in the hands and feet

1. Which of the following tests is used to diagnose sickle cell disease?

a) Blood smear

b) Electrocardiogram

c) MRI

d) CT scan

1. Which of the following medications is commonly used to manage severe pain in individuals with sickle cell disease?

a) Aspirin

b) Acetaminophen

c) Ibuprofen

d) Morphine

1. What is the primary mechanism of action of hydroxyurea in the management of sickle cell disease?

a) It increases the production of fetal hemoglobin

b) It stimulates red blood cell production

c) It reduces inflammation

d) It kills bacteria that can cause infections

1. Which of the following vaccines is recommended for individuals with sickle cell disease to help prevent infections?

a) Hepatitis A vaccine

b) Pneumococcal vaccine

c) Varicella vaccine

d) Measles, mumps, and rubella (MMR) vaccine

1. When is genetic counseling recommended for individuals with sickle cell disease?

a) When a couple is planning to have a child

b) After a diagnosis of sickle cell disease has been made

c) When a family member has been diagnosed with sickle cell disease

d) All of the above

PRE-POST TRAINING ANSWER SHEET

**Answer Sheet for Multiple Choice Questions (1-30)**

**Trainees name initials _________________________________**

**Age………….. Sex…………………………..**

**Cadre (e.g., MD, CO, AMO, RN etc) :_________________________________**

**Experience (Number of years )……………….**

**Department…………………**

**Instructions:**

To indicate your answer, circle the appropriate letter for each question. If you make a mistake, cross out the letter with a cross (X) and write the letter you want at the end of the row, for example:

| Question 1 |  | a | b | c | d |  | b |
| --- | --- | --- | --- | --- | --- | --- | --- |

**Multiple choice**

|  | A | B | C | D | E |
| --- | --- | --- | --- | --- | --- |
|  | A | B | C | D | E |
|  | A | B | C | D | E |
|  | A | B | C | D | E |
|  | A | B | C | D | E |
|  | A | B | C | D | E |
|  | A | B | C | D | E |
|  | A | B | C | D | E |
|  | A | B | C | D | E |
|  | A | B | C | D | E |
|  | A | B | C | D | E |
|  | A | B | C | D | E |
|  | A | B | C | D | E |
|  | A | B | C | D | E |
|  | A | B | C | D | E |
|  | A | B | C | D | E |
|  | A | B | C | D | E |
|  | A | B | C | D | E |
|  | A | B | C | D | E |
|  | A | B | C | D | E |
|  | A | B | C | D | E |
|  | A | B | C | D | E |
|  | A | B | C | D | E |
|  | A | B | C | D | E |
|  | A | B | C | D | E |
|  | A | B | C | D | E |
|  | A | B | C | D | E |
|  | A | B | C | D | E |
